# Plasma Levels of CXC Motif Chemokine 1 (CXCL1) and Chemokine 8 (CXCL8) as Diagnostic Biomarkers in Luminal A and B Breast Cancer

**DOI:** 10.3390/jcm11226694

**Published:** 2022-11-12

**Authors:** Joanna Motyka, Ewa Gacuta, Aleksandra Kicman, Monika Kulesza, Paweł Ławicki, Sławomir Ławicki

**Affiliations:** 1 Department of Population Medicine and Lifestyle Diseases Prevention, Medical University of Bialystok, 15-269 Bialystok, Poland; 2 Department of Gynecology and Gynecological Oncology, Medical University of Bialystok, 15-276 Bialystok, Poland; 3 Department of Aesthetic Medicine, Medical University of Bialystok, 15-267 Bialystok, Poland

**Keywords:** breast cancer, *adenocarcinoma ductale*, luminal A, luminal B, chemokines, CXCL1, CXCL8, CA 15-3, plasma concentration, *fibroadenoma*

## Abstract

Chemokines are involved in the regulation of immune balance and in triggering an immune response. CXCL1 and CXCL8 belong to the ELR-motif-containing group of CXC chemokines, which, in breast cancer (BC), stimulate angiogenesis and increase migration and invasiveness of tumor cells. The aim of this study was to evaluate CXCL1, CXCL8 and comparative marker CA 15-3 plasma concentrations in BC patients with luminal subtypes A and B. The study group consisted of 100 patients with BC, and the control group of 50 subjects with benign breast lesions and 50 healthy women. Chemokines concentrations were determined by ELISA method; CA15-3-by CMIA. Concentrations of CXCL8 and CA15-3 were significantly higher in BC total group and luminal B (for CA15-3 also in luminal A) subtype of BC than in healthy controls and subjects with benign lesions. In the total BC group, the highest SE, PPV and NPV were observed for CXCL8 (70%, 77.78%, 50%, resp.). A combined analysis of tested chemokines with CA 15-3 increased SE and NPV values (96%, 69.23%, resp.). The diagnostic power of the test (measured by area under ROC curve (AUC)) showed the highest value for CXCL8 in the total BC group (0.6410), luminal A (0.6120) and B subgroup of BC (0.6700). For the combined parameter, the AUC was increasing and reached the highest value for CXCL1 + CXCL8 + CA15-3 combination (0.7024). In light of these results, we suggest that CXCL8 could be used as an additional diagnostic marker that would positively influence the diagnostic utility of CA 15-3, especially in luminal B subtype of BC.

## 1. Introduction

Breast cancer is the most frequent malignancy affecting women, with more than 2 million new cases per year worldwide and more than 620,000 deaths. BC belongs to a highly heterogeneous group of cancers, which results in a great diversity in its development, course and response to treatment [1]. The key moment for every patient is the earliest possible diagnosis of a cancerous lesion and monitoring tits development. Current breast cancer diagnosis is mainly based on imaging, genetic and biochemical examinations. The implementation of screening mammography, improvements in systemic adjuvant therapy, and the introduction of targeted therapy led to a reduction in breast cancer mortality in developed countries against an ever-increasing number of newly diagnosed cases [2,3]. However, early detection of this cancer still remains a major challenge for health services. The diagnostic process is also aided by the determination of tumor markers in peripheral blood. The routine markers used for breast cancer are CA 15-3 and, less commonly, CA 27.29 or CEA, but because of their insufficient diagnostic sensitivity and specificity, especially in early stages of breast cancer, they are not sufficient to establish diagnosis. Nevertheless, CA 15-3 has found its use as a marker in predicting the course of the disease and the incidence of metastasis, and in monitoring the effectiveness of treatment [4]. Therefore, further research is being conducted to find markers whose concentrations could better correlate with the presence or stage of the disease. Such biomarkers are currently being sought among enzymes, hormones, chemokines and circulating RNA or DNA fragments [5,6,7].

The incidence of breast cancer is influenced by many interrelated factors, such as genetic and environmental factors, hormonal management, and lifestyle [7]. Other factors that have been found to predispose to cancer include chronic inflammation [8]. Disturbances in the expression of chemokines, as molecules involved in the regulation of inflammation, may contribute to an increased risk of breast cancer. They are also molecules that influence the process of carcinogenesis through interactions with chemokine receptors, directing cells towards proliferation, differentiation, invasion or metastasis. The overexpression of chemokines and their receptors has been demonstrated in various types of malignancies, including breast cancer [9,10]. CXCL1 and CXCL8 belong to the CXC chemokine family [10]. An abnormal expression of CXCL1 and CXCL8 has been found in many types of malignancies, including breast cancer. These chemokines are associated with the endothelial–neoplastic–matrix signaling network, regulation of inflammatory mediators, and interference in breast cancer development by controlling cell adhesion, angiogenesis, proliferation, migration and metastasis. Additionally, CXCL8 also enhances cancers by controlling stem cell mass, and CXCL1 is associated with the occurrence of drug resistance [11,12,13,14,15,16,17,18,19,20,21,22]. CXCL1 shows increased expression in the BC tumor stroma and its plasma levels have been linked to the number of circulating tumor cells [23,24]. Higher CXCL8 mRNA levels in breast cancer tissues were associated with significantly shorter overall survival [25]. CXCL8 is also overexpressed in breast cancer and positively correlates with inflammatory cell activity, which may account for the aggressiveness of these tumors [26].

In the present study, we investigated the plasma concentrations of CXCL1 and CXCL8 in female breast cancer patients as potential tumor markers in the diagnostic process, as an individual or combined parameter with the routinely used marker CA 15-3. In addition, we evaluated the concentrations of these chemokines among patients with benign breast lesions, as well as changes in plasma concentrations of the studied parameters before and after surgical treatment for BC patients.

## 2. Materials and Methods

We included a group of 100 patients with ductal adenocarcinoma breast cancer, *adenocarcinoma ductale* (BC-total), of luminal subtypes A and B, who underwent diagnosis and subsequent surgical treatment at the Bialystok Oncology Center in Poland. Histopathological evaluation and receptor status subtyping of breast lesions were performed at the hospital diagnostic stage on the basis of preoperative breast tumor biopsy or intraoperatively taken biopsy specimens. Patients for the study were divided into groups according to the receptor subtype into the subgroup of breast cancers with luminal subtype A (BC—Lum A) and the sub-group of breast cancers with luminal subtype B (BC—Lum B).

The control group consisted of 50 subjects with benign breast lesions, *fibroadenoma,* and 50 healthy women age-matched to the study. Detailed characteristics of studied groups are presented in Table 1.

The selection of the study and control groups, the preoperative and postoperative therapeutic management were carried out by the hospital unit in accordance with current clinical practice guidelines for the treatment of breast cancer. Patients with malignant lesions who received adjuvant preoperative treatment were excluded from the study. Patients with breast cancer underwent breast-conserving treatment with sentinel node evaluation or mastectomy, depending on the stage of the lesion. Pathomorphological assessment of the lesion included evaluation of the degree of malignancy and molecular features (evaluation of estrogen receptor (ER) and progesterone receptor (PR) expression, HER-2 receptor status and evaluation of Ki-67 proliferation index).

Postoperative evaluation of the concentrations of studied parameters was performed in the material obtained from BC patients 6–8 weeks after the performed surgical treatment, at the control moment before the introduction of further treatment with radiotherapy and/or chemotherapy.

We qualified patients with breast cancer or a benign lesion on the basis of gynecological examinations, followed by confirmatory examinations by the oncologist on the basis of imaging studies (mammography/USG/magnetic resonance imaging) and laboratory tests.

The healthy women included in the control group were volunteers who were qualified to participate in the study by a family doctor, and then a gynecologist of the University Clinical Hospital in Bialystok, Poland and participants of the Bialystok PLUS cohort study, in whom a detailed imaging diagnosis (abdominal ultrasound/magnetic resonance) and evaluation of laboratory results were performed, on the basis of which the gynecologist subsequently determined the possibility of inclusion in the study.

The study material was plasma obtained from venous blood collected for the anticoagulant lithium heparin. Venous blood was collected from the participants and centrifuged at 1810× *g* for 10 min. The centrifuged plasma was then pooled and stored at −85 °C until the day of the assay.

We measured plasma CXCL1 and CXCL8 concentrations with the use of immuno-enzymatic ELISA method (Quantikine ELISA Human CXCL1/GROα and Quantikine ELISA IL-8/CXCL8, R&D Systems Inc., Minneapolis, MN, USA). The assays were performed according to the manufacturer’s instructions provided with the kits, using double-sample determinations for the standard curve and tested samples. Intra-assay and inter-assay precision were determined by the manufacturer (CXCL1 2.4%, 4.7%; CXCL8 5.4%, 9.7%, respectively). For the measurement of CA 15-3 levels, we used a chemiluminescent microparticle immunoassay (CMIA) (Abbott, Chicago, IL, USA) according to the manufacturer’s protocols.

### Statistical Analysis

The analysis of obtained parameters was performed using PQStat ver.1.8.2 PQStat Software, (Poznan, Poland) and GraphPad Prism 9.3.1. for Windows, GraphPad Software, (San Diego, CA, USA).

After the evaluation of the normality of the distribution for the tested parameters with the Shapiro–Wilk test, which revealed significant deviations from the normal distribution, we performed statistical analysis using nonparametric tests. To assess statistical differences between two independent groups, we used the Mann–Whitney U test, whereas, when comparing between multiple groups, we used the Kruskal–Wallis’s test with the Conover–Iman post-hoc test. Comparisons between concentrations of studied parameters for paired measurements (preoperative period and 6–8 weeks since the procedure) were made with the Wilcoxon test for dependent pairs. Due to the insufficient number of premenopausal women in the study groups, we refrained from assessing the influence of this parameter on the obtained results. 

For the evaluation of the diagnostic features of the tested parameters: diagnostic sensitivity (SE), diagnostic specificity (SP), positive predictive value (PPV), negative predictive value (NPV), and diagnostic power, our analysis was performed on the basis of the area under the ROC curve (AUC) and the optimal cut-off points determined by the closest distance to corner method, which were, respectively: 34.44 pg/mL for CXCL1, 3.71 pg/mL for CXCL8 and 17.8 IU/mL for CA 15-3.

## 3. Results

### 3.1. Evaluation of Menopausal Status Influence on Constructed Groups

In order to assess the effect of the patients’ menopausal status on the analysis of the results in each group, we performed a comparison with the Mann–Whitney U test. Due to the low number of premenopausal patients, we decided to compare the individual groups selected throughout the study (BC-total group, luminal A and luminal B subgroups of BC, benign breast lesion group, healthy women group) with the same alternative groups, from which only patients with premenopausal status were excluded. This analysis did not reveal any significant differences between the analyzed groups (data not shown). However, we decided to perform an additional individual evaluation of the diagnostic features for only postmenopausal subjects of our research.

### 3.2. Preoperative Concentrations

**Figure 1:**
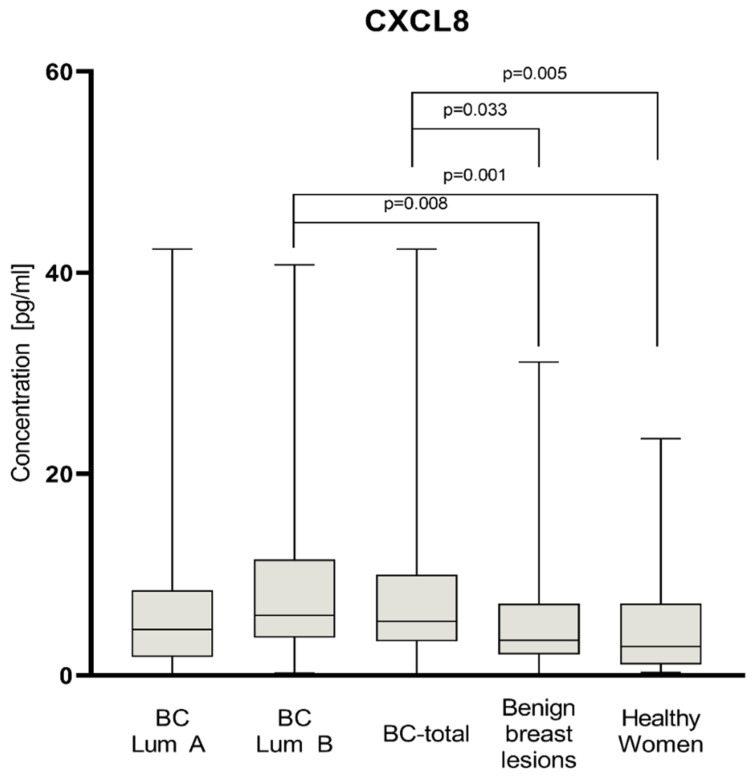
Plasma concentrations of CXCL8 of patients with BC (total group and subgroups), subjects with benign breast lesion and healthy women with highlighted statistically significant differences.

**Figure 2:**
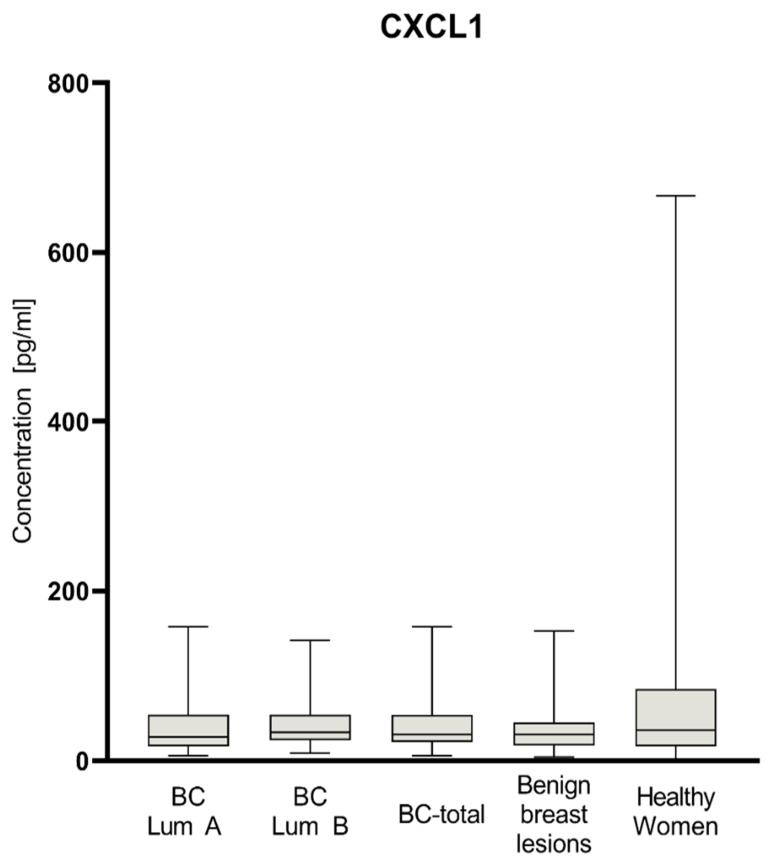
Plasma concentrations of CXCL1 of patients with BC (total group and subgroups), subjects with benign breast lesion and healthy women.

**Figure 3:**
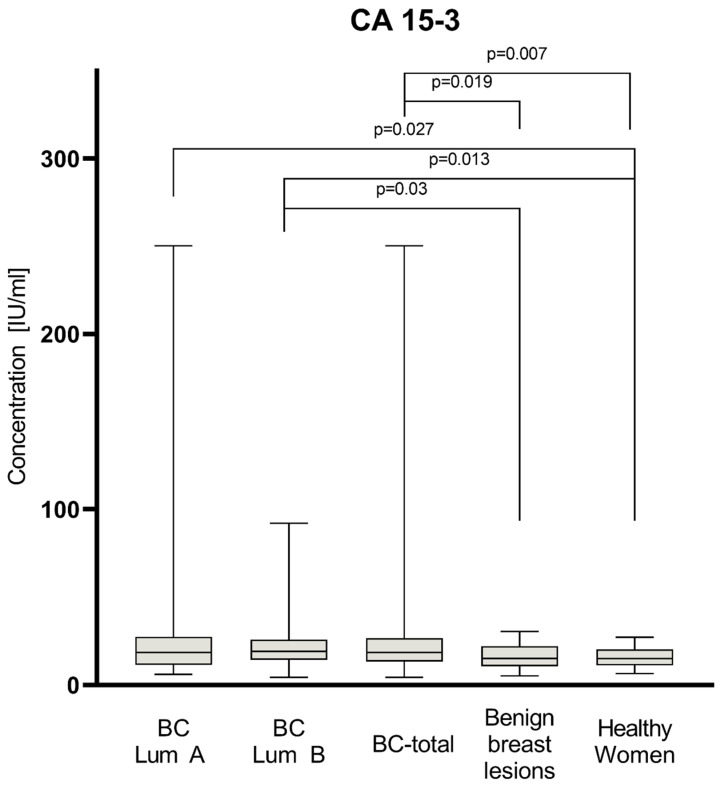
Plasma concentrations of CA 15-3 of patients with BC (total group and subgroups), subjects with benign breast lesion and healthy women, with statistically significant differences highlighted.

Plasma levels of tested parameters among all groups are presented in Figure 1, Figure 2 and Figure 3. Statistical analysis showed that BC-total patients had significantly higher concentrations of CXCL-8 (median 5.36 pg/mL) compared to healthy women (2.88 pg/mL, *p* = 0.005) and subjects with benign lesion (3.50 pg/mL, *p* = 0.033). Analyzing concentrations of comparative marker CA 15-3, we also noticed a higher concentration among all patients with BC (median 18.65 IU/mL) than among healthy women (15.05 IU/mL, *p* = 0.007), as well as among patients with benign lesion (15.2 IU/mL, *p* = 0.019).

In the group of BC patients with luminal subtype A, we found significantly higher concentrations for CA 15-3 (18.55 IU/mL) compared to the group of healthy women (*p* = 0.027). In the group of BC patients with luminal subtype B, concentrations of all parameters maintained the same relation as the BC-total group. CXCL8 concentrations were significantly higher in luminal B subtype of BC (5.95 pg/mL) than among healthy women (*p* = 0.001) and subjects with benign lesion (*p* = 0.008). CA 15-3 concentrations in patients with luminal B subtype of BC (19.2 IU/mL) were also significantly higher than in the group of healthy women (*p* = 0.013) and subjects with benign lesion (*p* = 0.03). CXCL1 did not show any significant statistical differences in concentrations in all study groups. In the BC group of patients with luminal subtype B, the median concentrations of CXCL8 (5.95 pg/mL), CXCL1 (33.55 pg/mL), and CA 15-3 (19.2 IU/mL) were higher than in the group of patients with luminal subtype A (4.57 pg/mL; 27.83 pg/mL; 18.55 IU/mL, respectively); however, the used test did not show these correlations as statistically significant.

**Figure 4:**
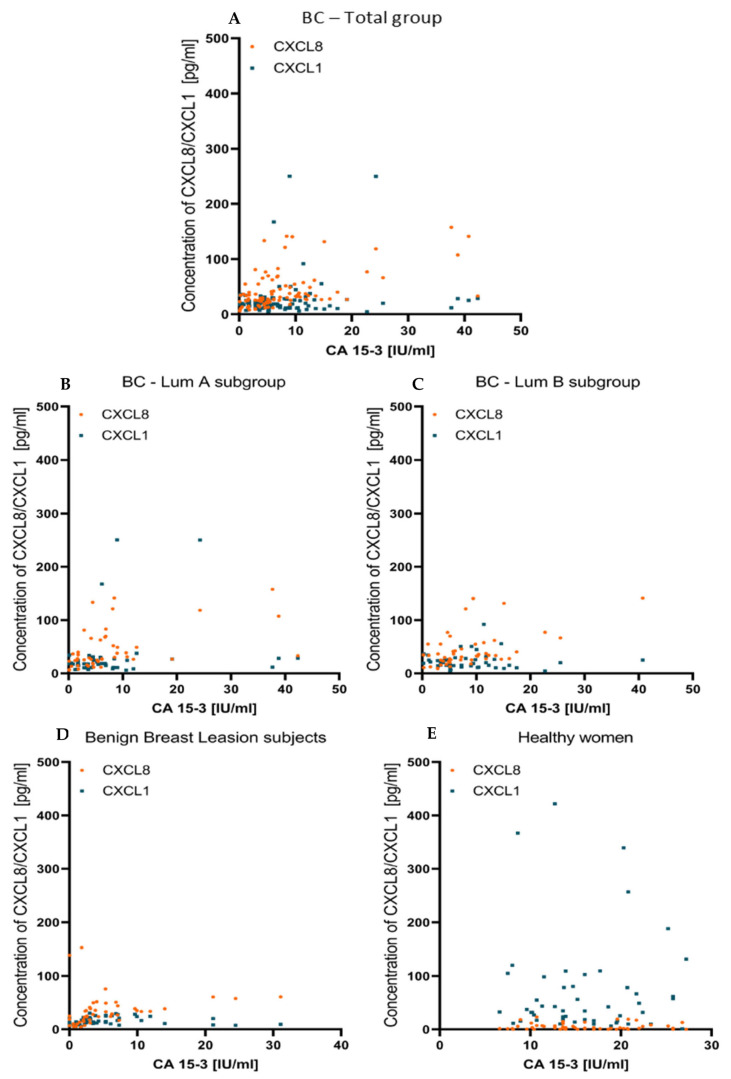
Correlation between preoperative level of tested chemokines and CA15-3, presented in the form of scatterplots in BC- total group (**A**), BC—Lum A subgroup (**B**), BC—Lum B subgroup (**C**), subjects with benign lesion (**D**) and healthy women (**E**).

Using r Spearman’s non-parametric test, we examined the correlations between the studied parameters, but we did not find any significant correlations between CXCL1, nor between CXCL8 and the comparative marker CA 15-3, in any of the study groups. Correlations between tested chemokines and CA 15-3 are reflected by scatterplots on Figure 4.

**Figure 5:**
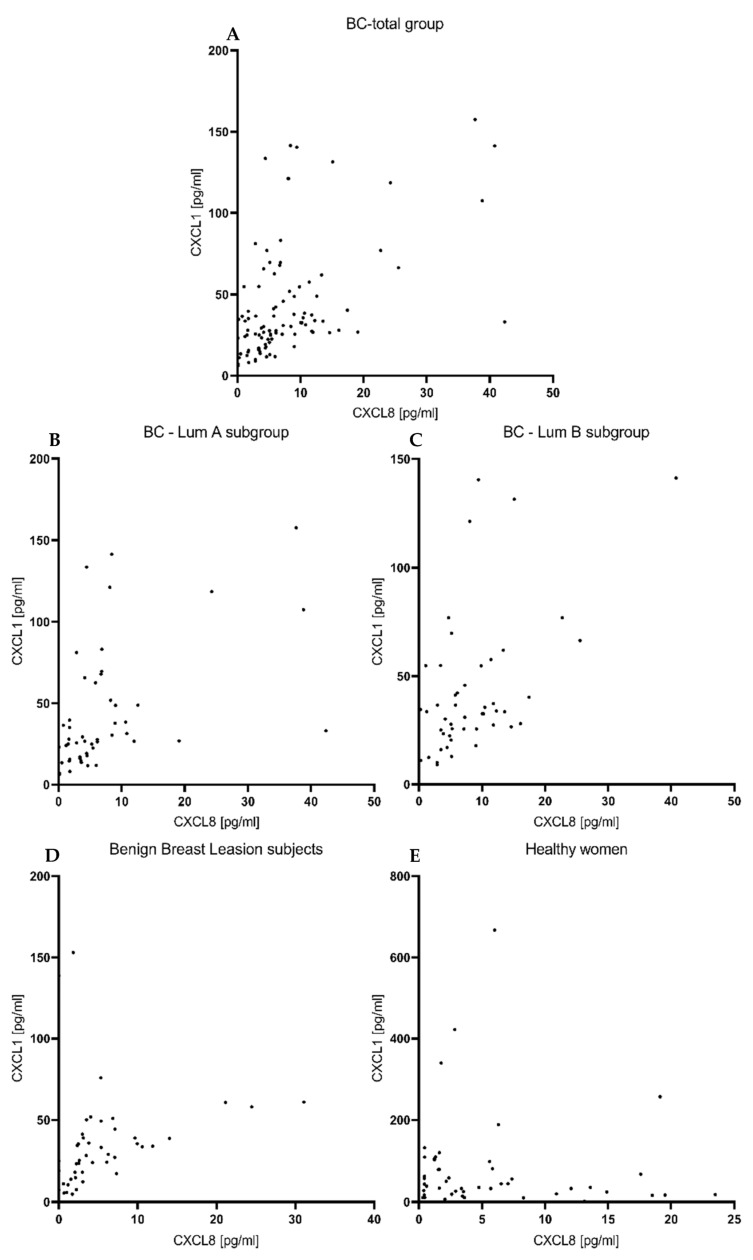
Correlation between CXCL1 and CXCL8, presented in the form of scatterplots in BC—total group (**A**), BC—Lum A subgroup (**B**), BC—Lum B subgroup (**C**), subjects with benign lesion (**D**) and healthy women (**E**).

However, we found significant correlations between CXCL1 and CXCL8 in the BC—total group (r = 0.5103 *p* < 0.001), both BC luminal subgroups—luminal A (r = 0.5701 *p* < 0.001) and luminal B (r = 0.4489, *p* = 0.001) and in the group of subjects with benign lesion (r = 0.5311, *p* < 0.001) (Figure 5).

### 3.3. Postoperative Concentrations

Our team was able to evaluate pre- and post-operation levels of the studied parameters among 43 of our patients (Table 2). At 4–6 weeks after the surgical treatment, a statistically significant decrease was observed, only for CA 15-3 (preoperative 18.2 IU/mL, postoperative 17.8 IU/mL, *p* = 0.029), when analyzing the whole pre-to-post-operative group. Statistical analysis performed on luminal A and luminal B subgroups no longer showed a significant reduction in CA 15-3 concentrations. Among patients of luminal A subtype of BC, statistical analysis also showed a significant increase in the concentrations of both chemokines tested in the postoperative period compared to the preoperative phase—CXCL1 increased from 30.832 pg/mL to 41.414 pg/mL (*p* = 0.048), while CXCL8 increased from 5.326 pg/mL to 7.229 pg/mL (*p* = 0.023). However, we did not show this relationship for either the subgroup of patients with luminal B BC or the entire study group.

In addition, we conducted a comparison between concentrations of studied parameters in patients after surgery against subjects with benign lesion and healthy women. In the total group of BC patients, CXCL8 remained at a higher level than for healthy women (*p* < 0.001) and subjects with benign lesion (*p* = 0.003). Additionally, for the luminal A and luminal B cancer patient subgroups, we showed significantly higher levels than for healthy women (luminal A *p* = 0.001; luminal B *p* = 0.011) and subjects with benign lesion (luminal A *p* = 0.006; luminal B *p* = 0.044). For CXCL1, we noticed significantly higher postoperative concentrations in the total BC group of patients only, compared to subjects with benign lesion (*p* = 0.005). Analyzing luminal A and B subgroups for CXCL1, we noticed adequately higher postoperative concentrations only, compared to subjects with benign lesion (luminal A *p* = 0.012; luminal B *p* = 0.042). By contrast, we did not observe significant differences in postoperative CA15-3 concentrations relative to the group of healthy women or subjects with benign lesion.

### 3.4. Diagnostic Criteria of CXCL1 and CXCL8–Pre- and Post-Menopausal Subjects

Table 3 contains the diagnostic criteria—SE, SP, PPV and NPV in BC patients.

The highest SE for the BC total patient group was achieved by CXCL8 (70%). The other parameters, CXCL1 and CA 15-3, had SE at similar levels (57%, 55%, respectively). All parameters reached fairly close SP values, with the highest value being reached for the comparative marker (CA 15-3—64%, CXCL8–60%, CXCL1—54%). An analysis of combined parameters increased SE of the tests for CXCL1 + CA 15-3 by up to 80%, and for CXCL8 + CA 15-3 by up to 88%, reaching the highest value for the combination of all tested factors—CXCL1 + CXCL8 + CA 15-3 up to 96%. When performing an analysis of combined parameters, we observed a decrease in SP.

Analyzing the individual subgroups, we noticed the similarity between the obtained SE results and the total group of patients. In both the Luminal A and Luminal B BC patient subgroups, the highest SE value for a single parameter was shown by CXCL8 (64%, 76%, respectively). In the Luminal A BC patient subgroup, the lowest SE value was recorded for CA 15-3 (54%), while, for the Luminal B BC patient subgroup, this was observed for CXCL1 (54%). When analyzing the combined parameters, the SE increased in both subgroups. In the Luminal A BC patient subgroup, the highest SE was shown by the combined of the three parameters CXCL1 + CXCL8 + CA 15-3 (98%), while in the Luminal B BC patient subgroup—for two combinations with equal values of 94%—CXCL8 + CA 15-3 and CXCL1 + CXCL8 + CA 15-3.

When examined as a total group of patients, CXCL8 was the only one of the tested chemokines to show a higher PPV (77.78%) and NPV (50%) than the comparative marker CA 15-3 (75.34%, 41.56%, respectively). Both features were the lowest for CXCL1 (71.25%, 38.59%, respectively). Analyzing the parameter combinations, we noticed an increase in NPV, with a relatively small decrease in PPV, reaching the following values for each combination: CXCL1 + CA 15-3—NPV 45.95%, PPV 70.8%, CXCL8 + CA 15-3—NPV 61.29%, PPV 73.95%, CXCL1 + CXCL8 + CA 15-3—NPV 69.23%, PPV 70.07%. The highest PPV, as a combined parameter, was achieved by the CXCL8 + CA 15-3 set (73.95%), while the highest NPV was achieved by CXCL1 + CXCL8 + CA 15-3 set (69.23%).

Looking at the subgroup of Luminal A BC patients, we recorded the highest PPV for CXCL8 (61.54%) as an individual parameter and the combination of two parameters CXCL8 + CA 15-3 (56.34%). Additionally, as a single parameter, CXCL8 had the highest NPV (62.5%). For the combined markers, the highest NPV was shown by the combination of all three compounds, CXCL1 + CXCL8 + CA 15-3 (90%), at the same time, with the lowest PPV of the combined sets (54.45%). In the Luminal B BC patient subgroup, CXCL8 also showed the highest PPV (65.52%) and NPV values (71.43%) for single markers. In the combined parameter analysis, the CXCL8 + CA 15-3 set demonstrated the highest NPV, with values of 86.36%.

**Figure 6:**
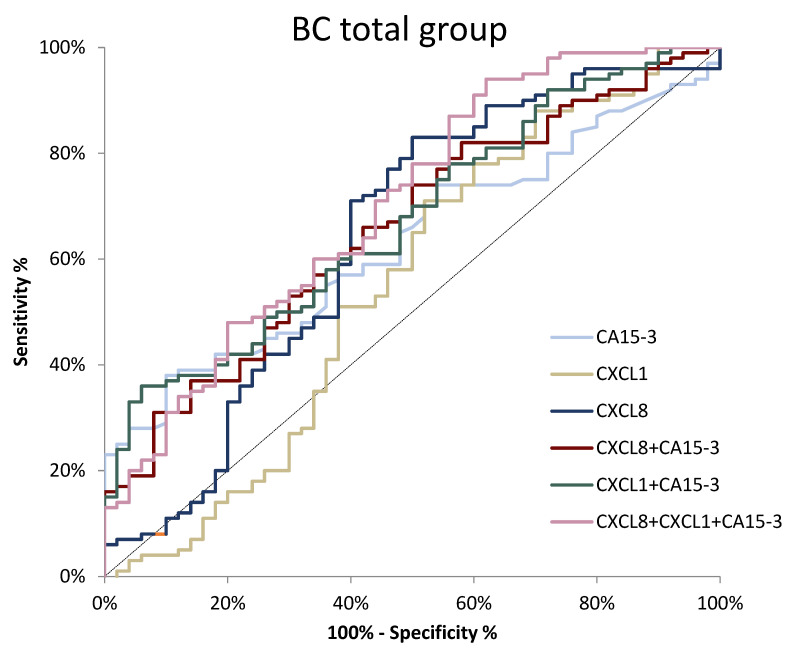
ROC curve for tested parameters in BC total group.

The ROC is a curve illustrating the dependence of SE on SP for the studied parameters, while the potential clinical utility as a tumor-marker will be demonstrated by the AUC, while also determining its diagnostic power. An AUC value of 0.5 is a borderline of the diagnostic usefulness of the test. The detailed parameters of the ROC curve analysis are shown in Table 4. In the total BC group, the AUC values for CXCL8 and CA 15-3 were significantly higher compared with AUC = 0.5 (*p* = 0.005, *p* = 0.010, respectively). The AUC for CXCL8 (0.6410) in the total BC group was higher than for CXCL1 (0.5496) and CA 15-3 (0.6300). Using a combination of CXCL1 or CXCL8 with CA 15-3 resulted in an increased AUC (0.6724, *p* = 0.001; 0.6582, *p* = 0.002, respectively). When using a combination of all tested compounds, we achieved the highest AUC of 0.7024 (*p* < 0.001) (Figure 6.).

**Figure 7:**
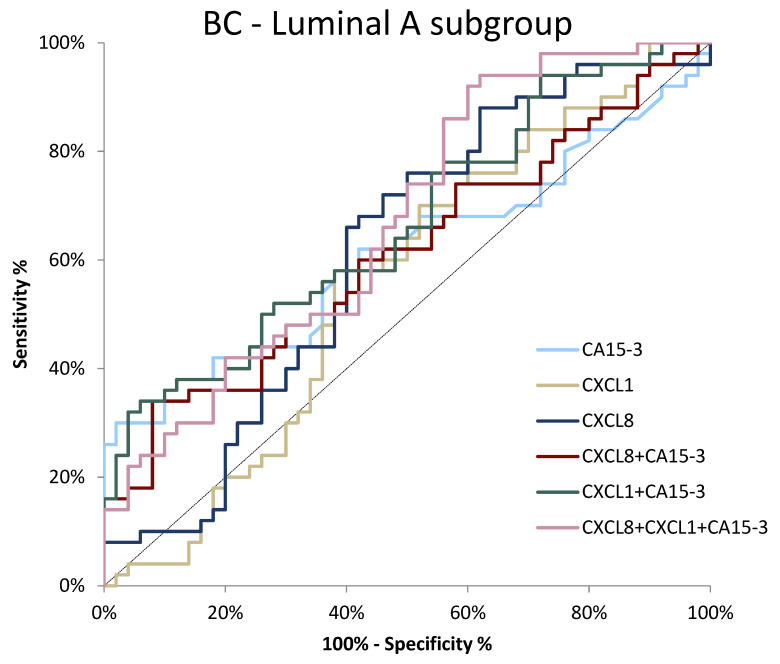
ROC curve for tested parameters in BC luminal A subgroup.

There was no single tested parameter that showed statistically significant diagnostic power (*p* > 0.05) in the BC luminal A subgroup of patients. However, the highest AUC values were observed for CXCL8 and CA 15-3 (0.6120, *p* = 0.052; 0.6114, *p* = 0.054, respectively). Among the combined parameters, the combination of both chemokines with comparative marker achieved the highest AUC value (0.6708, *p* = 0.002) (Figure 7).

**Figure 8:**
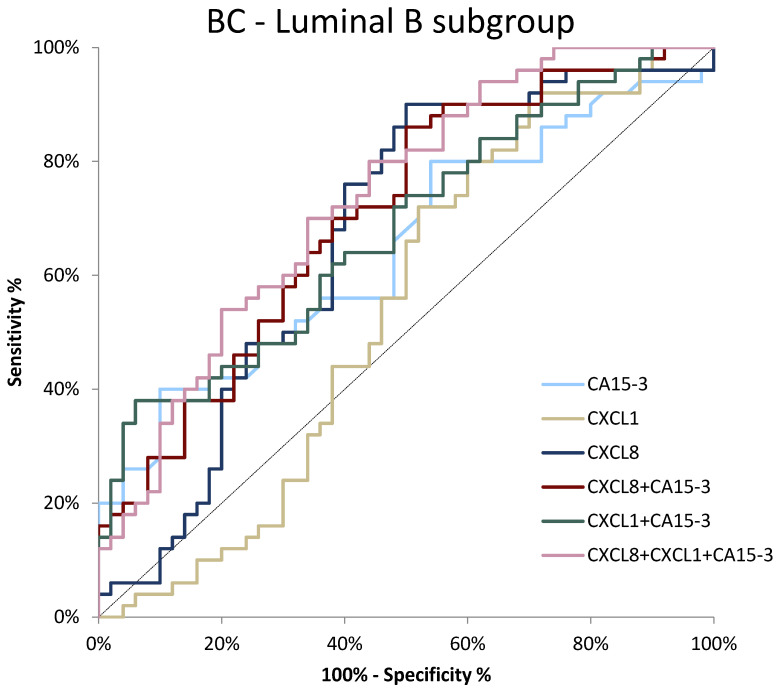
ROC curve for tested parameters in BC luminal B subgroup.

In the subgroup of luminal B BC patients, we observed the highest AUC once again for a combination of all tested parameters (0.7340, *p* < 0.001). Regarding the single analysis of the studied parameters, CXCL8 again showed the highest AUC (0.6700, *p* = 0.002), which exceeded the AUC values obtained for CA 15-3 and CXCL1 (0.6486, *p* = 0.007; 0.5424, *p* = 0.478, respectively). For CXCL1, the AUC values were not significantly higher compared to AUC = 0.5 (Figure 8).

A comparison of parameter combinations against the comparative marker CA 15-3 revealed that, in the total study group and the luminal A subgroup of BC, combining CA 15-3 with CXCL1 (*p* = 0.008) and CA15-3 with CXCL1 and CXCL8 (*p* = 0.002) significantly improved the quality of the test. In the luminal B subgroup, all combinations significantly improved the quality of the test (see Table 4).

### 3.5. Diagnostic Criteria of CXCL1 and CXCL8—Postmenopausal Subjects

Table 5 shows the diagnostic criteria of tested parameters, comparative marker and analyzed combinations of parameters calculated for the postmenopausal subjects of our study.

In a single-parameter analysis, CXCL8 showed the highest values for SE (68.89%), SP (65%), PPV (81.58%) and NPV (48.15%). The comparative marker CA 15-3 achieved identical sensitivity to CXCL8, while achieving lower values for the other diagnostic features—SE (54.44%), PPV (77.78%), NPV (38.81%). The lowest values of diagnostic features were demonstrated by CXCL1.

Combined parameter analysis increased the sensitivity of the test in each case, reaching the highest value for the combination of CXCL1 + CXCL8 + CA 15-3 (88.89%). However, combining parameters into panels resulted in a corresponding decrease in the SP value of the assay. Of the combined panels, the CXCL8 + CA 15-3 combination achieved the highest SP (37.5%), PPV (75.49%) and NPV (53.57%). The NPV for this combination also increased above the values achieved for all individual parameters.

Analyzing the results in the subgroups of luminal A and B BC patients, CXCL8 again reached the highest values for all diagnostic features—in luminal A subgroup—SE 65.12%; PPV 66.67%, NPV 63.41%; in luminal B subgroup SE 74.47%, PPV 71.43%, NPV 68.42%. Worse diagnostic features than CXCL8 in both luminal subgroups were demonstrated by CA 15-3 (luminal A SE 51.16%, PPV 61.11%, NPV 55.32%; luminal B SE 57.45%, PPV 65.85%, NPV 56.52%). Analogous to the results obtained for the whole group, CXCL1 in both luminal subgroups showed the worst values of the calculated criteria.

In luminal A cancer subgroup, the combined parameter analysis showed the highest SE (76.74%), SV (37.50%), PPV (56.9%) and NPV (60%) for the CXCL8 + CA 15-3 combination, reaching the highest SE and NPV values shown in the entire study. The three-parameter combination of CXCL1 + CXCL8 + CA 15-3 had an equally high SE value as the CXCL8 + CA 15-4 combination, while lower values were obtained for the other features.

In the subgroup of patients with luminal B BC, the combined analysis increased the SE and NPV of the tests, reaching the highest value of 100% for both features for the combination of CXCL1 + CXCL8 + CA 15-3. At the same time, this combination reached the lowest sensitivity recorded in the whole study (17.5%). Among the combined parameters, the CXCL8 + CA 15-3 combination showed the highest PPV (63.77%), but this value was lower than that obtained for the single parameters of both CXCL8 and CA 15-3.

The detailed parameters of the ROC curve analysis for only postmenopausal subjects of study are shown in Table 6.

In the total BC group, the AUC values for CXCL8 (0.6961) and CA 15-3 (0.6461) were significantly higher compared with AUC = 0.5 (*p* < 0.001, *p* = 0.008, respectively). The AUC for CXCL8 was the highest in the total BC group. Combining parameters into panels increased the AUC values for all parameters, reaching a peak for three-parametric combination CXCL1 + CXCL8 + CA 15-3 (0.7464, *p* < 0.001). The addition of a combination of parameters into panels led to a significant enhancement in the quality of the test compared to the single CA 15-3 marker (see Table 6).

When analyzing individual subgroups of BC, CXCL8 showed the highest values of AUC of all single parameters. In the luminal A subgroup of patients, only CXCL8 showed statistically higher AUC (0.6797, *p* = 0.005) compared to the AUC borderline value. Only a combination of all three parameters CXCL1 + CXCL8 + CA 15-3 (0.6901) exceeded the AUC value demonstrated for CXCL8. In the luminal A subgroup of BC patients, looking at acquired results of single parameters, the addition of CXCL1 to combined panels significantly enhanced the quality of the test.

In the luminal B subgroup of patients CXCL8 (0.7112), as well as CA 15-3 (0.6705), statistical importance was demonstrated compared to AUC = 0.5 (*p* < 0.001, *p* = 0.006, respectively). An analysis of combined parameters increased AUC values, which reached their peak value for a tree–parametric combination of CXCL1, CXCL8 and CA 15-3 (0.7883, *p* < 0.001). In the luminal B subgroup of patients, combined panels led to a significant enhancement in the quality of the test when compared to the individual CA 15-3 parameters.

## 4. Discussion

Breast cancer is one of the most common malignancies among women [1]. Many interrelated factors contribute to the incidence of BC, including genetic or environmental factors and hormonal imbalances [7,8]. According to the literature reports, the presence of chronic inflammation may also predispose to BC [8]. One of the mediators of the inflammatory process are chemokines. Additionally, chemokines are also involved in numerous adverse processes associated with cancer progression and invasion [9,10]. CXCL1 and CXCL8 have multidirectional effects, which can be associated with several adverse processes related to carcinogenesis, such as cell adhesion, angiogenesis, proliferation, migration, metastasis and the induction of resistance to pharmacological anticancer treatment [11,12,13,14,15,16,17,18,19,20,21,22]. The aforementioned properties of these chemokines translate into an unfavorable prognosis for cancer patients overexpressing these proteins [27,28].

Currently, medicine places great emphasis on early cancer diagnosis, which would enable faster treatment, resulting in improved patient prognosis. In many cancer groups, introduced screening programs, such as mammography, colonoscopy or smear tests, have improved the early detection of these diseases. However, additional methods for the early detection of cancer, especially at the asymptomatic stage, are constantly being sought [29,30]. Among the proposed diagnostic methods is the determination of circulating biomarkers [29]. In this study, we focused on evaluating the diagnostic utility of two chemokines, CXCL1 and CXCL8, as novel biomarkers in Luminal A and B breast cancer, as well as in control groups, i.e., patients with benign lesions (*fibroadenoma*) and healthy volunteers, alone and in correlation with the routinely measured breast cancer marker—CA 15-3.

CXCL1 is expressed in breast cancer cells and the stroma surrounding the cancerous lesion [18,24,31], and the expression levels of this chemokine depended on the size and stage of the tumor and the abundance of metastases [18]. In addition, the overexpression of CXCL1 in the stroma was associated with faster disease recurrence and worse patient prognosis [24]. In vitro, CXCL1 stimulated the proliferation, invasion, and migration of breast cancer cells while inhibiting apoptosis, which seems to partially explain the unfavorable prognosis of patients with overexpression of this chemokine [18,32]. At present, there are no studies determining plasma CXCL1 levels in patients with breast cancer. However, according to the literature data, elevated CXCL1 levels were found in patients with ovarian cancer, among others [33]. Conflicting results apply to lung cancer patients, where no differences were found between patients with an advanced stage of disease and healthy subjects. On the other hand, patients with early-stage cancer have lower levels of CXCL1 compared to advanced forms of the disease and healthy volunteers. However, it should be noted that the study was conducted on a small number of patients (*n* = 30, for each group) [34]. According to our results, there are no differences in plasma CXCL1 levels between patients with BC, benign lesions, and healthy women. However, it should be noted that breast cancer cells produce CXCL1 [31], but it is not known whether CXCL1 concentrations are high enough to exceed the detection threshold when determined by a given analytical method from peripheral blood samples. The results we obtained for CXCL1 are puzzling, as the concentrations by themselves showed no differences between the study groups; however, when analyzing diagnostic features, the inclusion of CXCL1 in the combined analysis decreased the level of specificity and both predictive values, while increasing the sensitivity of the test and significantly improving the quality of the test, especially in the luminal A subgroup of BC, whereas, in ROC analysis, CXCL8 showed no such effect. This relationship between the CXCL1 appeared both in the analysis of the entire study group and in the evaluation of postmenopausal patients only. Thus, it there seems to be some relationship between this parameter and breast cancer, but, to accurately determine the impact of this chemokine in diagnosis, it is necessary to determine the CXCL1 concentrations in patients with breast pathologies and healthy women on a larger and more diverse group of patients.

Similar to CXCL1, CXCL8 expression was demonstrated in BC tumor samples where mRNA levels were independent of tumor size and stage, metastatic abundance, and chemotherapy efficacy. In estrogen receptor-negative patients, high CXCL8 expression was associated with a worse prognosis for patients [25]. Consistent with our results, BC patients (of total BC group and luminal B subtype of BC group) had statistically higher serum CXCL8 levels than patients with benign lesions and healthy women. This agrees with the results obtained by Narita et al. [35]. Higher levels of CXCL8 in BC patients were also found by Benoy et al. [36], Celik et al. [37] and Ma et al. [32], but it should be noted that the analyses were performed in serum, not in plasma, as in our study. Additionally, according to Benoy et al. [36] and Ma et al. [32], CXCL8 concentrations correlated with cancer stage and the presence of metastatic foci. In our study, we did not perform an analysis of the relationship between CXCL8 concentrations and cancer stage and the presence of metastases. Unfortunately, in the present study, we did not analyze the dependence of CXCL8 levels on the stage of cancer and the presence of metastases; however, in future studies, we plan to perform an equal analysis in the plasma obtained from BC patients.

CA 15-3 is a marker that is routinely performed for the diagnosis of BC [4,38,39,40]. According to our results, CA 15-3 levels were higher in BC patients compared to the control and comparison groups. This agrees with previous studies conducted by our group [38,40]. It is notable that the pattern in statistical differences in CXCL8 concentrations in the study groups was identical to that shown by the comparative marker CA 15-3.

In our study, postoperative CXCL1 and CXCL8 concentrations in BC patients did not change significantly 4–6 weeks after surgical treatment. The rise in the luminal A subgroup of BC was most probably a response to the healing process of the surgical wound, especially since baseline concentrations in this receptor subtype were slightly lower than among patients with luminal subtype B of BC. To our knowledge, there are no studies on postoperative plasma CXCL8 concentrations in BC patients and CXCL1 in oncology patients. Wang et al. [41] evaluated the serum levels of CXCL8 before and after surgical treatment of patients with triple-negative BC and noted no change in its levels. However, we are unable to relate these results to those obtained by our team, since the paper does not take into account the postoperative cytokine-storm phase, which excludes CXCL8 testing immediately after surgical treatment [41]. However, according to Kumara et al. [42], in patients with colon cancer, high levels of CXCL8 persisted for 4 weeks, which partially agrees with the results we obtained. Within 4–6 weeks after surgery, we observed a decrease in CA 15-3 concentration, which agrees with the experience of Będkowska et al. [39].

In our study, we performed SE, SP, PPV and NPV assays to determine the diagnostic utility of the tested markers. The SE values for CXCL8 were higher than the SE determined for the routine marker CA 15-3. To the best of our knowledge, we are the first group to determine the diagnostic utility of CXCL1 and CXCL8 in BC and are, therefore, unable to relate our results to the work of other authors. However, it is worth noting that Pawluczuk et al. [43], in patients with gastric cancer, also obtained a higher SE value for CXCL8 compared to routinely determined marker CEA, although this was comparable to the SE for CA 19-9. In our study, we obtained a higher SP value for the routine marker CA 15-3 than for CXCL1 and CXCL8; still, all values were relatively close to each other. Interestingly, analyses performed in gastric cancer patients showed that CXCL8 has higher SP values than the routine marker (CA 19-9) [43].

The PPV and NPV values we obtained for CXCL8 were higher than those obtained for CA 15-3. When performing a combined analysis of tested parameters, NPV values increased, obtaining better numbers than for individual compounds. Pawluczuk et al. [43] had the same outcome in patients with gastric cancer, where the PPV and NPV determined for CXCL8 was higher than for the routine marker CA 19-9. Additionally, similarly to our data, performing a combined analysis increased NPV. It is unfortunate that no data exist on the diagnostic criteria of serum/plasma level of CXCL1 in oncology patients with breast cancer or other types of cancer [43].

The highest values of diagnostic power were observed for CXCL8, both in the total study group and in luminal subtype B of breast cancer. Additionally, values increased when analyzed together with CA 15-3. It should be noted that these results are novel and have not yet been evaluated by other authors worldwide regarding breast cancer. Team Pawluczuk et al. also evaluated the diagnostic significance of CXCL8 as a marker of gastric cancer. In analogy to our results, the AUC values of CXCL8 were superior to those obtained for classical tumor markers (for BC—CA 15-3, for gastric cancer—CEA and CA 19.9). In addition, we also noted the same pattern of improvement in AUC values by analyzing combined parameter panel (CXCL8 and CRP) [43].

Investigating circulating chemokines in the bloodstream is a new branch of our research cycle on diagnostic markers for breast cancer [38,39,40,44,45]. In light of our results, CXCL8 appears to provide the most improvements in the diagnostic process of breast cancer as a single parameter, as well as when combined with CA 15-3, but our study needs to be expanded. The group of patients we studied did not include patients with HER2-positive or triple-negative breast cancer, which are important subgroups because of its malignancy and difficulty in treating. Despite the fact that, in our study, the menopausal status of the patients did not have a major effect on the obtained results, this effect may also be influenced by the size of the study groups. Therefore, we do not exclude the possibility that, in a study of larger groups of patients, menopausal status may prove to be an important factor influencing the results. When performing our analysis, we based the statistics on tests that take into account the lack of normal distribution of the data. We observed a clear skewness towards higher concentrations. We also did not correct for outliers so as not to artificially modify the obtained results, especially since our groups consisted of only 100 patients and not, for example, 1000. Therefore, we know that there may be a possibility that our results will change if a greater number of patients and more types of breast cancer are analyzed. Nevertheless, we are the first group, to our knowledge, to analyze the diagnostic utility of CXCL1 and CXCL8 in breast cancer, in combination with CA 15-3, and we hope that our research will inspire other researchers and prove to be useful in the diagnostic process in the future.

## Figures and Tables

**Table 1:** Characteristics of examined groups: breast cancer (BC-total) group of patients with selected subgroups for analysis, comparison group of subjects with benign breast lesions and control group of healthy women.

Studied Group	Group Size	Menopausal Status		Age (Median) (Min–Max)	TNM Status (Number of Incidents)		Treatment (Number of Incidents)
		Premeno-Pausal	Postmeno-Pausal				
**Preoperative group of patients**							
Breast cancer total group:	100	11	89	60 (21–85)	T1N0M0 (51) T1cN0M0 (3) T1N0Mx (2) T1NxM0 (9) T1N1M0 (1)	T2N0M0 (24) T2NxM0 (4) T2N1M0 (4) T3N0M0 (1) T4N0M0 (1)	BCT + ALND (69) BCT (4) Amputation + ALND (27)
Luminal A	50	8	42	58 (21–81)	T1N0M0 (27) T1N0Mx (2) T1cN0M0 (2) T1NxM0 (7)	T2N0M0 (10) T2NxM0 (1) T3N0M0 (1)	BCT + ALND (37) Amputation + ALND (13)
Luminal B	50	3	47	62.5 (38–79)	T1N0M0 (24) T1cN0M0 (1) T1NxM0 (2) T1N1M0 (1)	T2N0M0 (14) T2NxM0 (3) T2N1M0 (4) T4N0M0 (1)	BCT + ALND (32) BCT (4)Amputation + ALND (14)
**Postoperative group of patients (matched pairs)**							
Breast cancer total group:	43	3	40	61 (35–81)	T1N0M0 (25) T1cN0M0 (1) T1N0Mx (1) T1NxM0 (4)	T2N0M0 (8) T2NxM0 (2) T2N1M0 (1) T4N0M0 (1)	BCT + ALND (33) Amputation + ALND (10)
Luminal A	22	1	21	62 (35–81)	T1N0M0 (13) T1N0Mx (1) T1cN0M0 (1)	T1NxM0 (3)T2N0M0 (4)	BCT + ALND (19) Amputation + ALND (3)
Luminal B	21	2	19	60 (38–79)	T1N0M0 (12) T1NxM0 (1) T2N0M0 (4)	T2NxM0 (2) T2N1M0 (1) T4N0M0 (1)	BCT + ALND (14) Amputation + ALND (7)
**Control groups**							
Benign breast lesion group	50	12	38	50 (22–83)	*Fibroadenoma*		N/A
Healthy women group	50	10	40	49.5 (25–69)	N/A		N/A

ALND—axillary lymph node dissection; BCT—breast-conserving treatment; N/A—not applicable.

**Table 2:** Plasma levels of tested parameters prior to and post to surgical treatment of breast cancer patients.

	CXCL1 [pg/mL] Median (Q1–Q3)	CXCL8 [pg/mL] Median (Q1–Q3)	CA 15-3 [IU/mL] Median (Q1–Q3)
	**BC—total group (*n* = 43)**		
Before surgery	32.606 (25.963–54.763)	7.261 (3.807–11.115)	18.2 (13.95–26.9)
After surgery (4–6 weeks)	40.254 (31.305–55.098)	7.313 (3.853–12.556)	17.8 (12.9–20.0)
*p*	0.059	0.443	**0.029**
	**BC—Luminal A subgroup (*n* = 22)**		
Before surgery	30.832(20.236–46.467)	5.326 (1.77–8.846)	18.25 (13.675–28.125)
After surgery (4–6 weeks)	41.414 (32.966–52.634)	7.229 (4.729–11.593)	18.100 (13.975–19.3)
*p*	**0.048**	**0.023**	0.153
	**BC—Luminal B subgroup (*n* = 21)**		
Before surgery	33.868 (27.363–57.562)	10.043 (5.153–12.286)	18.2 (14.6–25.9)
After surgery (4–6 weeks)	37.061 (29.965–81.177)	7.508 (2.73–12.579)	15.6 (12.4–20.1)
*p*	0.532	0.509	0.063

**Table 3:** Diagnostic criteria of tested parameters in patients with BC: total group and luminal A and B subgroups.

Tested Parameter	Diagnostic Criterium (%)	Breast Cancer		
		Lum A Subgroup	Lum B Subgroup	Total Group
CXCL1	SE	60	54	57
	SP	54	54	54
	PPV	56.6	54	71.25
	NPV	57.45	54	38.59
CXCL8	SE	64	76	70
	SP	60	60	60
	PPV	61.54	65.52	77.78
	NPV	62.50	71.43	50
CA 15-3	SE	54	56	55
	SP	64	64	64
	PPV	60	60.87	75.34
	NPV	58.18	59.26	41.56
CXCL1 + CA 15-3	SE	84	76	80
	SP	34	34	34
	PPV	56	53.52	70.8
	NPV	68	58.62	45.95
CXCL8 + CA 15-3	SE	80	94	88
	SP	38	38	38
	PPV	56.34	60.26	73.95
	NPV	65.52	86.36	61.29
CXCL1 + CXCL8 + CA 15-3	SE	98	94	96
	SP	18	18	18
	PPV	54.45	53.41	70.07
	NPV	90	75	69.23

SE—diagnostic sensitivity; SP—diagnostic specificity; PPV—positive predictive value; NPV—negative predictive value

**Table 4:** Characteristics of ROC curve for tested parameters in patients with BC: total group and luminal A and B subgroups.

Tested Parameter	AUC	SE (AUC)	95% C.I.	*p* (AUC = 0.5)	Comparison to CA 15-3 (*p*)
**Breast cancer—total group**					
CXCL1	0.5496	0.055	0.443–0.657	0.322	0.258
CXCL8	0.6410	0.052	0.539–0.742	**0.005**	0.874
CA 15-3	0.6300	0.045	0.541–0.719	**0.010**	**-**
CXCL1 + CA 15-3	0.6724	0.045	0.584–0.760	**0.001**	**0.008**
CXCL8 + CA 15-3	0.6582	0.046	0.568–0.749	**0.002**	0.066
CXCL1 + CXCL8 + CA 15-3	0.7024	0.046	0.612–0.793	**<0.001**	**0.002**
**Breast cancer—Luminal A subgroup**					
CXCL1	0.5568	0.059	0.412–0.672	0.334	0.516
CXCL8	0.6120	0.058	0.499–0.725	0.052	0.994
CA 15-3	0.6114	0.058	0.498–0.725	0.054	**-**
CXCL1 + CA 15-3	0.6648	0.054	0.559–0.771	**0.005**	**0.028**
CXCL8 + CA 15-3	0.6108	0.057	0.500–0.722	0.051	0.317
CXCL1 + CXCL8 + CA 15-3	0.6708	0.054	0.565–0.776	**0.002**	**0.041**
**Breast cancer—Luminal B subgroup**					
CXCL1	0.5424	0.060	0.425–0.660	0.478	0.185
CXCL8	0.6700	0.060	0.560–0.780	**0.002**	0.796
CA 15-3	0.6486	0.055	0.540–0.757	**0.007**	**-**
CXCL1 + CA 15-3	0.6800	0.053	0.576–0.784	**<0.001**	**0.025**
CXCL8 + CA 15-3	0.7056	0.052	0.604–0.807	**<0.001**	**0.036**
CXCL1 + CXCL8 + CA 15-3	0.7340	0.050	0.637–0.831	**<0.001**	**0.006**

**Table 5:** Diagnostic criteria of tested parameters in postmenopausal patients with BC: total group and luminal A and B subgroups.

Tested Parameter	Diagnostic Criterium (%)	Breast Cancer		
		Lum A Subgroup	Lum B Subgroup	Total Group
CXCL1	SE	44.19	42.55	43.33
	SP	42.50	42.50	42.50
	PPV	45.24	51.16	62.90
	NPV	41.46	38.64	25.00
CXCL8	SE	65.12	74.47	68.89
	SP	65.00	65.00	65.00
	PPV	66.67	71.43	81.58
	NPV	63.41	68.42	48.15
CA 15-3	SE	51.16	57.45	54.44
	SP	65.00	65.00	65.00
	PPV	61.11	65.85	77.78
	NPV	55.32	56.52	38.81
CXCL1 + CA 15-3	SE	69.77	78.72	74.44
	SP	27.50	27.50	27.50
	PPV	50.85	56.06	69.79
	NPV	45.83	52.38	32.35
CXCL8 + CA 15-3	SE	76.74	93.62	85.56
	SP	37.50	37.50	37.50
	PPV	56.90	63.77	75.49
	NPV	60.00	82.35	53.57
CXCL1 + CXCL8 + CA 15-3	SE	76.74	100.00	88.89
	SP	17.50	17.50	17.50
	PPV	50.00	58.75	70.80
	NPV	41.18	100.00	41.18

SE—diagnostic sensitivity; SP—diagnostic specificity; PPV—positive predictive value; NPV—negative predictive value

**Table 6:** Characteristics of ROC curve for tested parameters in postmenopausal patients with BC—total group and luminal A and B subgroups.

Tested Parameter	AUC	SE (AUC)	95% C.I.	*p* (AUC = 0.5)	Comparison to CA 15-3 (*p*)
**Breast cancer—total group**					
CXCL1	0.5633	0.060	0.446–0.680	0.250	0.288
CXCL8	0.6961	0.054	0.591–0.801	**<0.001**	0.515
CA 15-3	0.6461	0.049	0.550–0.742	**0.008**	**-**
CXCL1 + CA 15-3	0.6872	0.048	0.592–0.782	**<0.001**	**0.011**
CXCL8 + CA 15-3	0.7114	0.048	0.618–0.805	**<0.001**	**0.007**
CXCL1 + CXCL8 + CA 15-3	0.7464	0.048	0.652–0.841	**<0.001**	**<0.001**
**Breast cancer—Luminal A subgroup**					
CXCL1	0.5512	0.064	0.424–0.678	0.423	0.4631
CXCL8	0.6797	0.060	0.562–0.797	**0.005**	0.4813
CA 15-3	0.6195	0.062	0.497–0.742	0.061	**-**
CXCL1 + CA 15-3	0.6628	0.059	0.546–0.779	**0.011**	**0.050**
CXCL8 + CA 15-3	0.6471	0.060	0.529–0.766	**0.021**	0.079
CXCL1 + CXCL8 + CA 15-3	0.6901	0.058	0.576–0.804	**0.003**	**0.027**
**Breast cancer—Luminal B subgroup**					
CXCL1	0.5745	0.065	0.448–0.701	0.233	0.267
CXCL8	0.7112	0.058	0.581–0.825	**<0.001**	0.651
CA 15-3	0.6705	0.058	0.557–0.784	**0.006**	**-**
CXCL1 + CA 15-3	0.7059	0.055	0.598–0.814	**<0.001**	**0.029**
CXCL8 + CA 15-3	0.7601	0.052	0.659–0.861	**<0.001**	**0.003**
CXCL1 + CXCL8 + CA 15-3	0.7883	0.049	0.692–0.885	**<0.001**	**0.001**

## Data Availability

The data presented in this study are available on request from the corresponding author.

## References

[1] Sung H., Ferlay J., Siegel R.L., Laversanne M., Soerjomataram I., Jemal A., Bray F. (2021). Global Cancer Statistics 2020: GLOBOCAN Estimates of Incidence and Mortality Worldwide for 36 Cancers in 185 Countries. CA Cancer J. Clin..

[2] Azamjah N., Soltan-Zadeh Y., Zayeri F. (2019). Global Trend of Breast Cancer Mortality Rate: A 25-Year Study. Asian Pac. J. Cancer Prev..

[3] Swedish Organised Service Screening Evaluation Group (2006). Reduction in breast cancer mortality from the organised service screening with mammography: 2. Validation with alternative analytic methods. Cancer Epidemiol. Biomark. Prev..

[4] Duffy M.J., Evoy D., McDermott E.W. (2010). CA 15-3: Uses and limitation as a biomarker for breast cancer. Clin. Chim. Acta.

[5] Jafari S.H., Saadatpour Z., Salmaninejad A., Momeni F., Mokhtari M., Nahand J.S., Rahmati M., Mirzaei H., Kianmehr M. (2018). Breast cancer diagnosis: Imaging techniques and biochemical markers. J. Cell Physiol..

[6] Barzaman K., Karami J., Zarei Z., Hosseinzadeh A., Kazemi M.H., Moradi-Kalbolandi S., Safari E., Farahmand L. (2020). Breast cancer: Biology, biomarkers, and treatments. Int. Immunopharmacol..

[7] Houghton S.C., Hankinson S.E. (2021). Cancer Progress and Priorities: Breast Cancer. Cancer Epidemiol. Biomark. Prev..

[8] Shacter E., Weitzman S.A. (2002). Chronic inflammation and cancer. Oncology.

[9] Liu H., Yang Z., Lu W., Chen Z., Chen L., Han S., Wu X., Cai T., Cai Y. (2020). Chemokines and chemokine receptors: A new strategy for breast cancer therapy. Cancer Med..

[10] Bikfalvi A., Billottet C. (2020). The CC and CXC chemokines: Major regulators of tumor progression and the tumor microenvironment. Am. J. Physiol. Cell Physiol..

[11] Acharyya S., Oskarsson T., Vanharanta S., Malladi S., Kim J., Morris P.G., Manova-Todorova K., Leversha M., Hogg N., Seshan V.E. (2012). CXCL1 paracrine network links cancer chemoresistance and metastasis. Cell.

[12] Miyake M., Hori S., Morizawa Y., Tatsumi Y., Nakai Y., Anai S., Torimoto K., Aoki K., Tanaka N., Shimada K. (2016). CXCL1-mediated interaction of cancer cells with tumor-associated macrophages and cancer-associated Fibroblasts Promotes Tumor Progression in Human Bladder Cancer. Neoplasia.

[13] Xu J., Zhang C., He Y., Wu H., Wang Z., Song W., Li W., He W., Cai S., Zhan W. (2012). Lymphatic endothelial cell-secreted CXCL1 stimulates lymphangiogenesis and metastasis of gastric cancer. Int. J. Cancer..

[14] Wang Z., Wang Z., Li G., Wu H., Sun K., Chen J., Feng Y., Chen C., Cai S., Xu J. (2017). CXCL1 from tumor-associated lymphatic endothelial cells drives gastric cancer cell into lymphatic system via activating integrin β1/FAK/AKT signaling. Cancer Lett..

[15] Kuo P.L., Shen K.H., Hung S.H., Hsu Y.L. (2012). CXCL1/GROα increases cell migration and invasion of prostate cancer by decreasing fibulin-1 expression through NF-κB/HDAC1 epigenetic regulation. Carcinogenesis.

[16] Han K.Q., He X.Q., Ma M.Y., Guo X.D., Zhang X.M., Chen J., Han H., Zhang W.W., Zhu Q.G., Zhao W.Z. (2015). Targeted silencing of CXCL1 by siRNA inhibits tumor growth and apoptosis in hepatocellular carcinoma. Int. J. Oncol..

[17] Wang L., Zhang C., Xu J., Wu H., Peng J., Cai S., He Y. (2016). CXCL1 gene silencing inhibits HGC803 cell migration and invasion and acts as an independent prognostic factor for poor survival in gastric cancer. Mol. Med. Rep..

[18] Ma K., Yang L., Shen R., Kong B., Chen W., Liang J., Tang G., Zhang B. (2018). Th17 cells regulate the production of CXCL1 n breast cancer. Int. Immunopharmacol..

[19] Wang Y., Liu J., Jiang Q., Deng J., Xu F., Chen X., Cheng F., Zhang Y., Yao Y., Xia Z. (2017). Human Adipose-Derived Mesenchymal Stem Cell-Secreted CXCL1 and CXCL8 Facilitate Breast Tumor Growth By Promoting Angiogenesis. Stem Cells.

[20] Mishra A., Suman K.H., Nair N., Majeed J., Tripathi V. (2021). An updated review on the role of the CXCL8-CXCR1/2 axis in the progression and metastasis of breast cancer. Mol. Biol. Rep..

[21] Al-Khalaf H.H., Al-Harbi B., Al-Sayed A., Arafah M., Tulbah A., Jarman A., Al-Mohanna F., Aboussekhra A. (2019). Interleukin-8 Activates Breast Cancer-Associated Adipocytes and Promotes Their Angiogenesis- and Tumorigenesis-Promoting Effects. Mol. Cell Biol..

[22] Singh J.K., Simões B.M., Howell S.J., Farnie G., Clarke R.B. (2013). Recent advances reveal IL-8 signaling as a potential key to targeting breast cancer stem cells. Breast Cancer Res..

[23] Divella R., Daniele A., Savino E., Palma F., Bellizzi A., Giotta F., Simone G., Lioce M., Quaranta M., Paradiso A. (2013). Circulating levels of transforming growth factor-βeta (TGF-β) and chemokine (C-X-C motif) ligand-1 (CXCL1) as predictors of distant seeding of circulating tumor cells in patients with metastatic breast cancer. Anticancer Res..

[24] Zou A., Lambert D., Yeh H., Yasukawa K., Behbod F., Fan F., Cheng N. (2014). Elevated CXCL1 expression in breast cancer stroma predicts poor prognosis and is inversely associated with expression of TGF-β signaling proteins. BMC Cancer.

[25] Fang Q.I., Wang X., Luo G., Yu M., Zhang X., Xu N. (2017). Increased CXCL8 Expression Is Negatively Correlated with the Overall Survival of Patients with ER-Negative Breast Cancer. Anticancer Res..

[26] Chavey C., Bibeau F., Gourgou-Bourgade S., Burlinchon S., Boissière F., Laune D., Roques S., Lazennec G. (2007). Oestrogen receptor negative breast cancers exhibit high cytokine content. Breast Cancer Res..

[27] Yu S., Yi M., Xu L., Qin S., Li A., Wu K. (2020). CXCL1 as an Unfavorable Prognosis Factor Negatively Regulated by DACH1 in Non-small Cell Lung Cancer. Front. Oncol..

[28] Cao Z., Fu B., Deng B., Zeng Y., Wan X., Qu L. (2014). Overexpression of Chemokine (C-X-C) ligand 1 (CXCL1) associated with tumor progression and poor prognosis in hepatocellular carcinoma. Cancer Cell Int..

[29] Lokshin A., Bast R.C., Rodland K. (2021). Circulating Cancer Biomarkers. Cancers.

[30] Hunter B., Hindocha S., Lee R.W. (2022). The Role of Artificial Intelligence in Early Cancer Diagnosis. Cancers.

[31] Yang C., Yu H., Chen R., Tao K., Jian L., Peng M., Li X., Liu M., Liu S. (2019). CXCL1 stimulates migration and invasion in ER negative breast cancer cells via activation of the ERK/MMP2/9 signaling axis. Int J. Oncol..

[32] Ma Y., Ren Y., Dai Z.J., Wu C.J., Ji Y.H., Xu J. (2017). IL-6, IL-8 and TNF-α levels correlate with disease stage in breast cancer patients. Adv. Clin. Exp. Med..

[33] Bolitho C., Hahn M.A., Baxter R.C., Marsh D.J. (2010). The chemokine CXCL1 induces proliferation in epithelial ovarian cancer cells by transactivation of the epidermal growth factor receptor. Endocr. Relat. Cancer.

[34] Spaks A., Jaunalksne I., Spaka I., Chudasama D., Pirtnieks A., Krievins D. (2015). Diagnostic Value of Circulating CXC Chemokines in Non-small Cell Lung Cancer. Anticancer Res..

[35] Narita D., Seclaman E., Anghel A., Ilina R., Cireap N., Negru S., Sirbu I.O., Ursoniu S., Marian C. (2016). Altered levels of plasma chemokines in breast cancer and their association with clinical and pathological characteristics. Neoplasma.

[36] Benoy I.H., Salgado R., Van Dam P., Geboers K., Van Marck E., Scharpé S., Vermeulen P.B., Dirix L.Y. (2004). Increased serum interleukin-8 in patients with early and metastatic breast cancer correlates with early dissemination and survival. Cli Cancer Res..

[37] Celik B., Yalcin A.D., Genc G.E., Bulut T., KulogluGenc S., Gumuslu S. (2016). CXCL8, IL-1β and sCD200 are pro-inflammatory cytokines and their levels increase in the circulation of breast carcinoma patients. Biomed. Rep..

[38] Piskór B.M., Przylipiak A., Dąbrowska E., Sidorkiewicz I., Niczyporuk M., Szmitkowski M., Ławicki S. (2020). Plasma Level of MMP-10 May Be a Prognostic Marker in Early Stages of Breast Cancer. J. Clin. Med..

[39] Będkowska G.E., Gacuta E., Zbucka-Krętowska M., Ławicki P., Szmitkowski M., Lemancewicz A., Motyka J., Kobus A., Chorąży M., Paniczko M. (2021). Plasma Levels and Diagnostic Utility of VEGF in a Three-Year Follow-Up of Patients with Breast Cancer. J. Clin. Med..

[40] Piskór B.M., Przylipiak A., Dąbrowska E., Sidorkiewicz I., Niczyporuk M., Szmitkowski M., Ławicki S. (2021). Plasma Concentrations of Matrilysins MMP-7 and MMP-26 as Diagnostic Biomarkers in Breast Cancer. J. Clin. Med..

[41] Wang R.X., Ji P., Gong Y., Shao Z.M., Chen S. (2020). Value of CXCL8-CXCR1/2 axis in neoadjuvant chemotherapy for triple-negative breast cancer patients: A retrospective pilot study. Breast Cancer Res. Treat..

[42] Shantha Kumara H.M.C., Sutton E., Bellini G.A., Yan X., Cekic V., Gandhi N.D., Whelan R.L. (2018). Plasma interleukin-8 levels are persistently elevated for 1 month after minimally invasive colorectal resection for colorectal cancer. Mol. Clin. Oncol..

[43] Pawluczuk E., Łukaszewicz-Zając M., Gryko M., Kulczyńska-Przybik A., Mroczko B. (2021). Serum CXCL8 and Its Specific Receptor (CXCR2) in Gastric Cancer. Cancers.

[44] Dąbrowska E., Przylipiak A., Zajkowska M., Piskór B.M., Sidorkiewicz I., Szmitkowski M., Ławicki S. (2020). Possible Diagnostic Application of CXCL12 and CXCR4 as Tumor Markers in Breast Cancer Patients. Anticancer Res..

[45] Zajkowska M., Gacuta E., Kozłowska S., Lubowicka E., Głażewska E.K., Chrostek L., Szmitkowski M., Pawłowski P., Zbucka-Krętowska M., Ławicki S. (2019). Diagnostic power of VEGF, MMP-9 and TIMP-1 in patients with breast cancer. A multivariate statistical analysis with ROC curve. Adv. Med. Sci..

